# Research on Three-Axis Vibration Characteristics and Vehicle Axle Shape Identification of Cement Pavement Under Heavy Vehicle Loads Based on EMD–Energy Decoupling Method

**DOI:** 10.3390/s25134066

**Published:** 2025-06-30

**Authors:** Pengpeng Li, Linbing Wang, Songli Yang, Zhoujing Ye

**Affiliations:** 1 National Center for Materials Service Safety, University of Science and Technology Beijing, Beijing 100083, China; 2 School of Environmental, Civil, Agricultural and Mechanical Engineering, College of Engineering, University of Georgia, Athens, GA 30602, USA

**Keywords:** cement concrete pavement, acceleration, intercorrelation analysis, triaxial signals, low-speed heavy loads

## Abstract

**Highlights:**

This study develops a MEMS-based triaxial vibration monitoring system to capture dynamic pavement responses under heavy-duty vehicle loads. Using empirical mode decomposition (EMD) and short-time energy (STE) analysis, this research study identifies distinct energy peaks associated with axle impacts, with vertical (*Z*-axis) vibrations showing the highest amplitude and frequency components. A robust method for accurate axle identification is proposed, overcoming signal noise interference and offering significant contributions to pavement health monitoring and traffic management systems.

**What are the main findings?**

- Vertical (*Z*-axis) vibrations have the highest amplitude and frequency components, highlighting their key role in pavement response.
- EMD and STE analyses effectively identify axle impacts and enable accurate axle configuration recognition.

**What is the implication of the main finding?**

- The findings contribute to better pavement health monitoring and performance assessment under heavy vehicle loads.
- Provides a reliable method for axle identification, supporting infrastructure management and optimizing traffic flow.

**Abstract:**

The structural integrity of cement concrete pavements, paramount for ensuring traffic safety and operational efficiency, faces mounting challenges from the escalating burden of heavy-duty vehicular traffic. Precise characterisation of pavement dynamic responses under such conditions proves indispensable for implementing effective structural health monitoring and early warning system deployment. This investigation examines the triaxial dynamic response characteristics of cement concrete pavement subjected to low-speed, heavy-duty vehicular excitations, employing data acquired through in situ field measurements. A monitoring system incorporating embedded triaxial MEMS accelerometers was developed to capture vibration signals directly within the pavement structure. Raw data underwent preprocessing utilising a smoothing wavelet transform technique to attenuate noise, followed by empirical mode decomposition (EMD) and short-time energy (STE) analysis to scrutinise the time–frequency and energetic properties of triaxial vibration signals. The findings demonstrate that heavy, slow-moving vehicles generate substantial triaxial vibrations, with the vertical (*Z*-axis) response exhibiting the greatest amplitude and encompassing higher dominant frequency components compared to the horizontal (X and Y) axes. EMD successfully decomposed the complex signals into discrete intrinsic mode functions (IMFs), identifying high-frequency components (IMF1–IMF3) associated with transient vehicular impacts, mid-frequency components (IMF4–IMF6) presumably linked to structural and vehicle dynamics, and low-frequency components (IMF7–IMF9) representing system trends or ambient noise. The STE analysis of the selected IMFs elucidated the transient nature of axle loading, revealing pronounced, localised energy peaks. These findings furnish a comprehensive understanding of the dynamic behaviour of cement concrete pavements under heavy vehicle loads and establish a robust methodological framework for pavement performance assessment and refined axle load identification.

## 1. Introduction

In recent years, spurred by economic expansion, the scale of infrastructure development within traffic engineering has continuously broadened. This growth has precipitated a sharp increase in the demand for substantial-tonnage and heavy-bridge trucks, normalizing the phenomenon of “heavy load” in road transportation. Conventional pavement types include cement concrete and asphalt concrete. Cement concrete pavement is widely adopted for high-capacity highways due to its superior attributes, such as elevated stiffness, significant load distribution capability, robust bending and wear resistance, and commendable stability [1,2]. Numerous investigations demonstrate that the excessive dynamic loads imposed by heavy vehicles represent a significant contributor to the early onset of damage and fatigue fracture in cement concrete pavements. The dynamic forces exerted by heavy vehicles induce distresses such as cracks, potholes, and differential settlement in cement concrete pavements, which progressively escalate into pavement deterioration or even extensive damage, simultaneously compromising the ride quality, comfort, and safety of transporting vehicles [3,4]. Consequently, studying the dynamic response of cement pavement subjected to heavy vehicle excitation provides a foundational basis for structural design and traffic load management [5,6].

The global proliferation of intelligent transportation systems (ITSs), fuelled by advancements in electronics and computing, offers effective solutions to traffic challenges such as congestion. Real-time traffic surveillance is pivotal to ITSs, with precise vehicle axle identification playing a critical role in optimizing traffic flow, evaluating infrastructure integrity, and integrating autonomous vehicles. Current techniques for axle identification are broadly classified as embedded and non-embedded. Embedded systems utilize sensors (e.g., strain gauges, electromagnetic sensors, fibre optics) to capture the road’s response to vehicular loading, from which features are extracted for vehicle classification [7,8]. Non-embedded systems primarily employ image processing of video or infrared data. However, video-based systems are vulnerable to environmental variables (rain, snow, dirt) and variations in camera positioning and angle. While microwave radar technology [9] furnishes information regarding distance, speed, and azimuth, its accuracy can be impaired by the Doppler effect [10] and necessitates costly recalibration [11]. Inductive loop technology [12,13] represents another established ITS technique. This methodology requires embedding cables within the road surface, generating measurable currents upon vehicle passage for the determination of speed and wheelbase. Nevertheless, installation is disruptive and maintenance is expensive.

Dynamic Weigh-in-Motion (WIM) systems are extensively employed for axle detection, providing measurements of weight, axle count, wheelbase, and speed [14,15]. However, their reliance on piezoelectric quartz sensors limits their performance under electromagnetic interference, and their substantial cost and installation constraints impede widespread deployment. Fiber Bragg grating (FBG)-based WIM systems present a viable alternative, as evidenced by researchers such as Al-Tarawneh et al. [16], who developed an FBG-based WIM system, and Malla et al. [17], who applied fibre optic sensors to assess wheel loads on highways. Batenko et al. [18] further explored the potential of fibre optic sensors in WIM systems, analysing factors influencing measurement accuracy. Despite these advancements, the inherent fragility of fibre optic structures and the high cost associated with the demodulation equipment restrict their large-scale adoption [19].

In the investigation of pavement dynamic response, while extensive research exists on asphalt pavement dynamic loading, studies addressing the impact of heavy vehicles on cement concrete pavements are comparatively limited. Although both are categorized as rigid pavements, their differing material properties, design principles, and structural configurations necessitate distinct dynamic analysis methodologies. Li et al. [20] investigated the time–frequency characteristics of cement concrete pavement dynamic response at varying vehicle speeds, observing signal attenuation. Optimal sensor spacing is paramount, given the direct proportionality between vibration signals and speed and the substantial attenuation observed. Ricardo et al. [21] employed a U-Net-based semantic segmentation technique and deep learning to detect pavement cracks, enhancing accuracy when integrated with vibration data. Dong et al. [22] developed a self-sensing cementitious sensor utilizing carbon nanofibers, demonstrating precise detection of vehicle speed and pedestrian movement, thereby advancing intelligent pavement technology. Peng et al. [23] explored the dynamic response of various concrete girder bridges subjected to asphalt pavement vibratory compaction through field tests and 3D finite element analysis, recommending tailored vibration parameters for optimal structural safety. Zhao et al. [24] proposed a methodology for calculating cement concrete pavement loads and thermal stresses utilizing FWD and GPR data, significantly improving the accuracy of fatigue stress calculations. Cai et al. [25] conducted cyclic vibration tests on a cement-amended railway embankment, analysing dynamic load distribution and attenuation under varying axle loads, speeds, and rainfall. At the same time, many scholars have studied the vibration signal of vehicle-road coupling system by numerical simulation [26]. Luo et al. [27] utilized ABAQUS to model cement concrete panel vibration, identifying the foundation reaction coefficient and void area as key factors influencing the fundamental frequency, providing insights for future intelligent pavement inspection. Zuzulova et al. [28] investigated the shear and pullout resistance of transfer bars under dynamic loading, highlighting their practical applications. Overall, current research predominantly centres on indoor, vibration-based assessments of cement concrete panels. Further investigation into triaxial vibration response under vehicular excitation is needed to elucidate the dynamic evolution mechanisms under heavy traffic conditions.

Presently, research concerning the dynamic response of cement concrete pavement primarily concentrates on stress, strain, and uniaxial vibration characteristics, with fewer studies addressing triaxial vibration characteristics and their interactions. Existing studies often rely on controlled indoor experiments, lacking long-term monitoring data under authentic road vehicle loads. Many studies exclusively focus on frequency domain analysis, neglecting in-depth discussions on time–frequency characteristics, energy distribution, and multi-modal decomposition. In summary, to comprehensively explore the vibration response characteristics of cement concrete pavement subjected to heavy-duty vehicles, this paper introduces the development of a pavement vibration monitoring system. By embedding a three-axis acceleration sensor within the pavement structure, it facilitates the monitoring of pavement vibration response under heavy vehicles, enabling the analysis of time-domain characteristics and frequency composition of cement concrete pavement vibration signals. This provides essential data and a foundational basis for the performance evaluation and structural design of heavy-duty cement pavement.

## 2. Field Tests of Subgrade Soil Under Heavy-Duty Vehicle Load

### 2.1. Three-Axis Vibration Monitoring System

**Figure 1:**
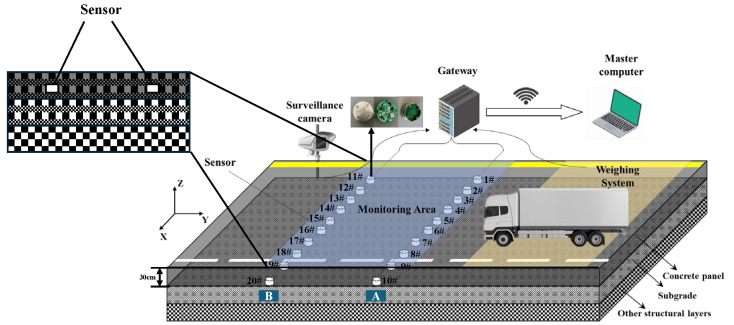
Schematic diagram of the sensor layout.

The field test was conducted on an experimental section of highway cement concrete pavement in Yunnan Province, featuring a 30 cm thick concrete slab. During the pavement laying process, acceleration sensors were synchronously embedded to establish a three-axis dynamic response monitoring system for collecting pavement structural dynamic signals under heavy vehicle traffic. The pavement structure design of this experimental section is designed to bear heavy-load traffic, and the design parameters of each structural layer, such as subgrade, are shown in Table 1 below:

The three-axis pavement vibration monitoring system comprises acceleration sensors, a gateway, and a main control computer. The cylindrical acceleration sensor package transmits data via a CAN bus to the gateway, which in turn relays the collected raw data to the master computer for real-time display and storage via network transmission. The experimental road section, with a width of 4.1 m, had a total of 20 sensors installed vertically across the traffic direction. These were arranged in two groups of 10 sensors each, with the groups spaced 3 m apart. Within each group, adjacent sensors were spaced 0.3 m apart. The first and last sensors in each group were positioned 0.4 m from the edge of the road. All sensors were buried at a depth of 2 cm below the pavement surface. The system deployment following sensor burial is illustrated in Figure 1. Through preliminary experiments with vibration signal response features at different depths, a 2 cm depth was ultimately chosen for sensor installation. This depth allows for the avoidance of direct wear on the road surface while still being sufficiently close to the point of load application. It enables the capture of vibration signals with high signal-to-noise ratio and rich details, thus preventing excessive signal attenuation and dispersion, which can occur with deeper burial. For directional reference, the lateral direction of the road surface is designated as the X-direction, the traffic flow direction as the Y-direction, and the direction perpendicular to the road surface as the z-direction.

The primary hardware components of the acceleration sensor, depicted in Figure 1, include a Printed Circuit Board (PCB), a stainless steel housing, data lines, and a data transfer interface. The PCB serves as the core element of this sensor. Its main constituents are an ultra-low-power CPU, a MEMS acceleration sensor, a buck regulator chip, and a CAN communication chip. Specifically, the MEMS acceleration sensor is a triaxial accelerometer with a measurement range of ±2 g. Its digital output signal undergoes calculation and processing by the CPU before being ultimately transmitted via the CAN communication chip. The sensor has a diameter of 40 mm and a thickness of 15 mm. Encased in a stainless steel shell, it exhibits high compressive strength, ensuring reliable operation under demanding conditions such as high load, repeated impacts, and hot and humid environments.

### 2.2. Pavement Vibration Response Signal Acquisition

The field data acquisition is mainly divided into two parts: vibration signal acquisition and road video shooting, and the axle weight data of the vehicle is obtained through the weighing system. The experimental steps are as follows:

(1) The performance of the sensors is critical for the experimental research. All sensors must be calibrated before being installed on-site, and comprehensive laboratory tests are conducted for their performance and stability;
(2) Check the monitoring system line connection, pre-test to confirm that all sensor data acquisition function is normal;
(3) Set the sampling frequency of 500 Hz following the initiation of data acquisition, while starting the camera to shoot the road vehicle information;
(4) After the rear wheels of the vehicle pass through the 1# sensor arrangement array, wait for 30 s to click the End button and save the collection;
(5) Synchronise the video, weighing system, and vibration data storage.

Based on the relevant research and preliminary experimental test results, the vibration frequencies induced by vehicular excitation on cement pavements are typically concentrated below 200 Hz. According to the Nyquist–Shannon sampling theorem, a sampling frequency of 500 Hz (more than twice the highest target frequency) is sufficient to accurately capture the key dynamic characteristics of the signal, prevent signal aliasing, and effectively manage the data volume for convenient storage and processing. In this paper, a total of 70 sets of road dynamics response data under heavy traffic were collected, and the collected data were analysed and studied according to the three indexes of axle, axle weight, and speed in the next section. Based on differences in load and axle type, typical data from 2–80 t vehicles were summarized in the experiment as shown in Table 2, and the three-axle monitoring data were analysed by taking the second group of vehicle experiments with a load of 21.8 t as an example.

**Figure 2:**
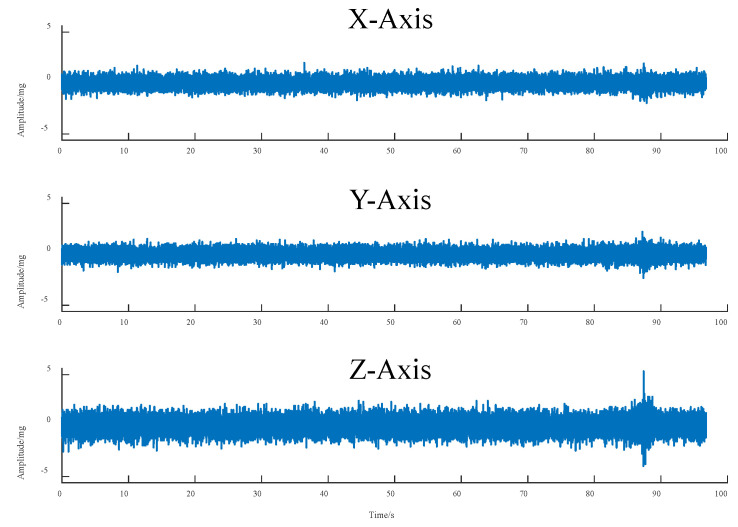
Original three-axis vibration signal.

Figure 2 illustrates the road vibration data recorded as a two-axle vehicle, with a load capacity of 21.8 t, traverses the monitoring point. Concurrent video analysis confirms that the peak signals align precisely with the vehicle’s passage, unequivocally establishing that the peak vibration signals at the monitoring point are indeed attributable to the excitation from the test vehicle. As is evident in Figure 2, the triaxial vibration response signals captured by the monitoring system are substantially corrupted by noise. Notably, the number and magnitude of signal fluctuations in the X- and Y-directions are considerably greater than those in the Z-direction. The power response is most pronounced along the *Z*-axis. The presence of these noise signals introduces considerable interference, complicating both the identification of vehicle excitation events and the subsequent isolation and analysis of power response signals. Consequently, denoising the raw signal is a necessary prerequisite.

## 3. Methods and Principles of Data Analysis

This section is divided into subheadings. It provides a concise and precise description of the experimental results, their interpretation, as well as the experimental conclusions that can be drawn.

### 3.1. Signal Noise Reduction Based on Smooth Wavelet Transform

The main sources of noise include the far-field environmental vibrations generated by vehicles on other lanes, noise introduced due to the imperfect coupling between sensors and the road surface, as well as electrical noise from the data acquisition system. Therefore, the noise reduction processing of data is crucial for subsequent data analysis and processing. The smooth wavelet transform is used to reduce noise in the raw vibration signals collected. The smooth wavelet transform (SWT), which overcomes the problem of translational sensitivity of the discrete wavelet transform (DWT), utilizes smooth and continuous wavelet functions to effectively analyse the signal or image, retaining the important information and filtering out the unwanted interferences, which can decompose the signal to different scales, facilitating the processing of the noise with different frequency components. The SWT does not perform down-sampling operations during the decomposition process, so its coefficients are the same as the original signal length. Therefore, its decomposed coefficients have the same length as the original signal, maintaining translation invariance. The decomposition process of the SWT can be expressed by the following equation:

(1) $$W_{j,k}=\sum_{l}h_{j,l}W_{j-1,k-2^{j-1}l}$$

(2) $$V_{j,k}=\sum_{l}g_{j,l}W_{j-1,k-2^{j-1}l}$$

where W_j,k_ denotes the detail coefficients of the jth layer, V_j,k_ denotes the approximation coefficients of the jth layer, and h_j,l_ and g_j,l_ denote the high-pass and low-pass filter coefficients, respectively.

After comparing the signal-to-noise ratio (SNR) effects of wavelet denoising using different wavelet basis functions [19], this paper ultimately selected db4 as the wavelet basis function, set the decomposition level to 5, and adopted the rigrsure adaptive threshold selection rule (for threshold selection) to achieve a balance between denoising and signal detail preservation.

**Figure 3:**
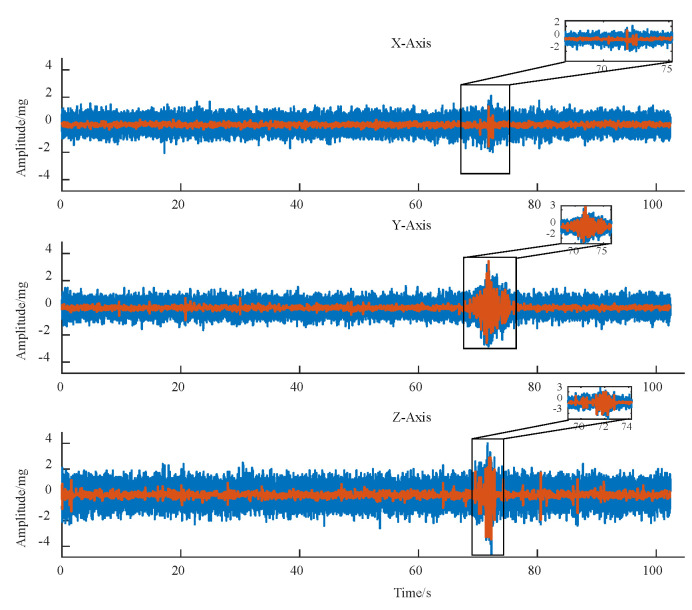
Comparison chart of noise reduction effect.

In this section, a three-axle truck with a load capacity of 75.8 t and a speed of 20 km/h was selected to analyse the road surface response signal collected when driving through the monitoring point. First, it adopted the above-mentioned noise reduction method to reduce the noise in the monitoring data. Then, it obtained the three-axle power response time curve of the road surface shown in Figure 3 after de-trending and smoothing. The smooth wavelet noise reduction method was used to reduce and smooth the three-axis vibration signal, and the comparison before and after noise reduction is shown in Figure 3. As a result, the denoised signal retains most of the details of the original signal while effectively eliminating signal burrs.

### 3.2. EMD Decomposition

The original vibration signal is complex and consists of noise signals and axle excitation signals, where the axle excitation signals are mainly concentrated in specific frequency bands, while the noise signals are uniformly distributed over the frequency range. In order to eliminate the noise and extract the useful signal, the empirical modal decomposition (EMD) method is used for signal reconstruction [29,30,31,32,33,34,35]. It is a decomposition method that decomposes the original signal into several intrinsic components, i.e., intrinsic modal functions (IMFs). Each IMF represents a detailed component of the original signal in a different frequency band. The IMF is a function that satisfies the following two requirements:

(1) The difference between the number of zero crossing points and the number of extreme points of the IMF is at most one throughout the data series;
(2) The local mean of the IMF at any moment should be zero.

In summary, the process of decomposing the aforementioned vibration signal f(t) is as follows:

First, all local maxima and local minima are identified from the original signal f(t).

Let t_i_ be the location of the local maxima or minima. The point of extreme value is

(3) $$t_{i}:\;f\left(t_{i}\right)>f\left(t_{i-1}\right)\;and\;f\left(t_{i}\right)>f\left(t_{i+1}\right)$$

The point of minimal value is

(4) $$t_{j}:\;f\left(t_{j}\right)<f\left(t_{j-1}\right)\;and\;f\left(t_{j}\right)<f\left(t_{j+1}\right)$$

These local maxima and local minima are then used to construct the upper and lower envelopes, respectively. The cubic spline interpolation method is usually used to connect these extreme points to obtain the upper envelope e_up_(t) and the lower envelope e_low_(t) envelope of the signal.

The average of the upper and lower envelopes, m(t), is considered the local trend of the signal:

(5) $$m_{1}\left(t\right)=\frac{e_{up}\left(t\right)+e_{low}\left(t\right)}{2}$$

A new signal h_1_(t) is obtained by removing the local average trend from the original signal using equation:

(6) $$h_{1}\left(t\right)=f\left(t\right)-m_{1}\left(t\right)$$

Removing this IMF from the original signal, the remaining signal is

(7) $$r_{1}\left(t\right)=f\left(t\right)-{IMF}_{1}\left(t\right)$$

At this point, r_1_(t) is the portion of the signal that is lower in frequency than the original signal, representing the low-frequency characteristics and trends of the signal.

The EMD decomposition is continued for the remaining signal r_1_(t), and the above steps are repeated to extract the next IMF_2_(t) through the process defined by

(8) $$r_{2}\left(t\right)=r_{1}\left(t\right)-{IMF}_{2}\left(t\right)$$

This is repeated until the residual signal r_n_(t) no longer contains any local extremes or becomes monotonic. After several EMD decompositions, the original signal f(t) can be expressed as a linear combination of the individual IMF components and the final residual r(t):

(9) $$f\left(t\right)=\sum_{i=1}^{n}\;{\mathrm{IMF}}_{i}\left(t\right)+r_{n}\left(t\right)$$

IMF_i_(t) is the i-th eigenmode function, representing localized oscillations at different time scales.

r_n_(t) is the residual of the decomposition, usually the trend or constant part of the original signal.

Empirical mode decomposition (EMD), as a powerful adaptive signal processing method, can decompose a signal based on its own time-scale characteristics without requiring any preset basis functions. However, it also has limitations, such as mode mixing, where a single Intrinsic Mode Function (IMF) may contain vastly different time scales, or signals of the same time scale are distributed across different IMF components, which can compromise the reliability of subsequent feature extraction. Nevertheless, the core objective of this paper was to conduct a preliminary investigation into the feasibility of applying the EMD method to process pavement vibration signals for extracting vehicle axle-type features. Furthermore, the impact response signal generated when a vehicle passes over the sensor is characterized by strong transient properties and a concentrated dominant frequency. These relatively distinct signal characteristics reduce the risk of severe mode mixing to a certain extent. Therefore, the standard EMD method was chosen for this study. In future research, we will consider introducing more robust decomposition methods, such as CEEMDAN, to further enhance the accuracy and stability of feature extraction.

Regarding parameter selection, the stopping criteria used in the EMD decomposition process directly affect the decomposition results. This study followed the classic criteria proposed by Huang et al., which ensures that each IMF component satisfies its definition through an iterative sifting process. Based on the literature review and by optimizing for the best decomposition results from our experimental data, we set the stopping threshold for the sifting iterations to 0.2 to ensure a balance between preventing over-decomposition and maintaining the orthogonality of the IMFs.

In order to verify the effectiveness of the empirical mode decomposition (EMD) method, a set of synthetic signal experiments containing three sine waves with different frequencies was designed in this paper. The specific experimental process was as follows:

Firstly, three basic signal components with different frequency characteristics were constructed: (1) a low-frequency signal f_1_(t) = 2sin (πt), with a frequency of 0.5 Hz, which represents the low-frequency trend of the signal;

(2) An intermediate frequency signal f_2_(t) = sin (6πt), with a frequency of 3 Hz, representing the intermediate frequency oscillation characteristics of the signal;

(3) A high-frequency signal f_3_(t) = 0.5 sin (24πt), with a frequency of 12 Hz, reflecting the high-frequency detail characteristics of the signal.

These three basic signals are superimposed to obtain a composite signal, x(t), defined as follows:

x(t) = f_1_(t) + f_2_(t) + f_3_(t). (10)

**Figure 4:**
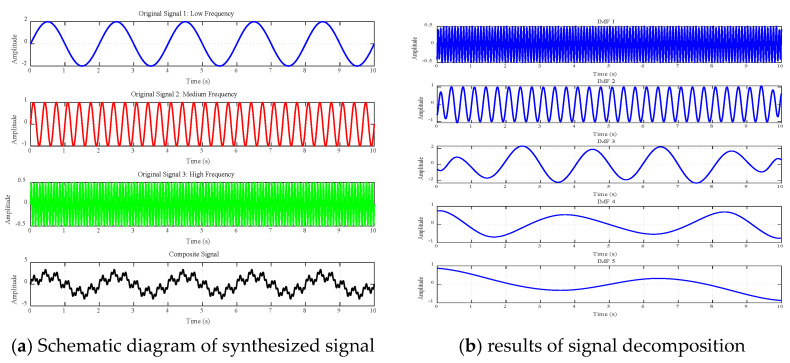
Schematic diagram of the EMD decomposition of the signal.

The composite signal is decomposed by EMD method, and a series of eigenmode functions (IMF) are obtained. According to the spectrum characteristics of environmental noise and vibration data under actual working conditions, this paper selected low-, medium-, and high-frequency bands of signals to synthesize new signals and then used the EMD method to decompose the synthesized signals. Figure 4. shows the original signal, the synthesized signal, and their EMD decomposition results. It can be clearly observed from the figure that the EMD method successfully separates the signal components with different frequency characteristics, and the frequency of each IMF component decreases from high to low, which is consistent with the basic principle of EMD decomposition. The experimental results confirm the effectiveness of the EMD method in signal decomposition and feature extraction.

**Figure 5:**
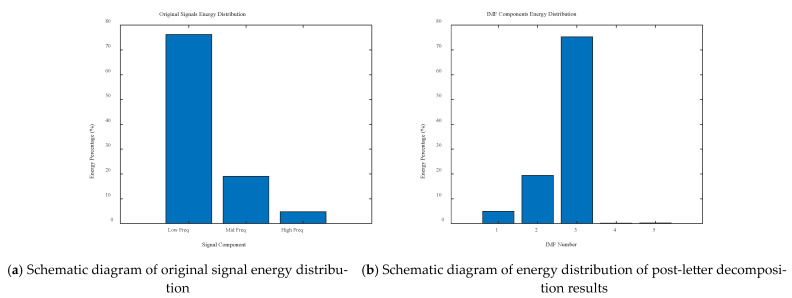
Signal energy analysis.

It is worth noting that the EMD decomposition yields a greater number of IMFs (5) than the number of original signal components (3). This is due to the adaptive nature of the EMD method, where subsequent IMF components may contain residual trends in the signal or components arising from boundary effects. In order to determine the effective frequency components, the original signal and IMF signal energy were analysed, and the results are shown in Figure 5. In the original signal, the energy share of the low-frequency signal f1 is 76.19%, the energy share of the medium-frequency signal f2 is 19.05%, and the energy share of the high-frequency signal f3 is 4.76%. In the EMD decomposition results, the energy share of IMF1 is 4.92%, the energy share of IMF2 is 19.40%, the energy share of IMF3 is 75.25%, the energy share of IMF4 is 0.17%, and the energy share of IMF5 is 0.26%. The first three IMF components contain the main energy of the signal, which is in accordance with the composition of the original signal, so IMF1-IMF3 are the effective components of signal decomposition.

In order to qualitatively verify the similarity between the EMD-decomposed IMF components and the original function, correlation coefficients, were calculated for analysis. The results show that the correlation coefficient between the first IMF component and the original high-frequency signal (f_3_) is 0.996, that of the second IMF component and the mid-frequency signal (f_2_) is 0.993, and that of the third IMF component and the low-frequency signal (f_1_) is 0.9975. These high correlations indicate that the EMD method successfully separates the signal components at different frequencies. The experimental results show that the EMD method can effectively decompose the composite signal into components with different frequency characteristics.

### 3.3. Short-Term Energy Analysis

From the previous section, it can be found that the vehicle-induced vibration signals are characterized by non-stationary time-varying characteristics, and the statistical characteristics of the signals (e.g., mean, variance, spectrum, etc.) vary with time. Traditional frequency domain analysis methods (e.g., Fourier transform) are unable to deal with the signal characteristics effectively. However, by dividing the signal into small time windows, each of which can be approximated as smooth, the short-time energy analysis method can effectively capture the time-varying characteristics, local fluctuations, and the nonsmoothness of the signal, which is very important for improving the accuracy, diagnostic capability, and real-time performance of signal analysis.

Short-time energy (STE) is a commonly used feature in signal processing to analyse the energy distribution of nonsmooth signals over time. Typically, the energy of a signal is characterized by fluctuations over time, which is especially significant in application scenarios such as speech signals and sensor data [36,37,38,39,40,41]. The short-time energy can reflect the instantaneous energy magnitude of a signal at a specific moment in time, which helps to capture the local features of the signal.

For a discrete signal x[n], the short-time energy is given by

(11) $$E_{m}=\sum_{n=0}^{N-1}\;w[n]\cdot |x[mR+n]{|}^{2}$$

where

E_m_ is the short-time energy of the m-th frame;

x[n] is a discrete signal;

w[n] is a window function (usually a rectangular window or a Hanning window);

N is the window size (i.e., frame length);

R is the frame shift (i.e., inter-frame step size);

mR + n is the specific position of each frame in the signal sequence.

(12) $$\begin{array}{c} E_{x}[m]=\sum_{n=0}^{N-1}\;w[n]\cdot |x[mR+n]{|}^{2}\\ E_{y}[m]=\sum_{n=0}^{N-1}\;w[n]\cdot |y[mR+n]{|}^{2}\\ E_{z}[m]=\sum_{n=0}^{N-1}\;w[n]\cdot |z[mR+n]{|}^{2} \end{array}$$

## 4. Discussion

In order to further understand the characteristics of the vibration signals of cement pavements under on-board excitation, in addition to the basic analysis of the three-axis time and frequency domains, it is also necessary to carry out a more detailed study from the perspective of nonlinear and nonsmooth signals. Since the vibration signals caused by vehicle excitation often contain complex dynamic responses, and their characteristics may change significantly with time and frequency, it is difficult to fully reveal their intrinsic characteristics and energy distribution laws by only relying on the traditional time–frequency domain analysis. Empirical mode decomposition (EMD), as an adaptive method for nonlinear and nonsmooth signal processing, can decompose the original vibration signals into a series of intrinsic mode functions (IMFs), which provide a powerful tool for studying the local characteristics of the signals. Based on this, the following section uses the EMD method to decompose the triaxial vibration signal, analyse the characteristics of different modal components, and discuss them in depth in conjunction with the short-time energy. This can not only reveal the distribution law and time-varying characteristics of different frequency bands of energy in the vibration signal but also provide a key basis for the subsequent anomaly identification and damage diagnosis in the health monitoring of pavement structures. Therefore, this part of this study extends the dimension of the characterization of vibration signals and also provides an important support for the mining of multi-scale characteristics of vibration signals

### 4.1. Three-Axis Characteristics of Vibration Response Signals

**Figure 6:**
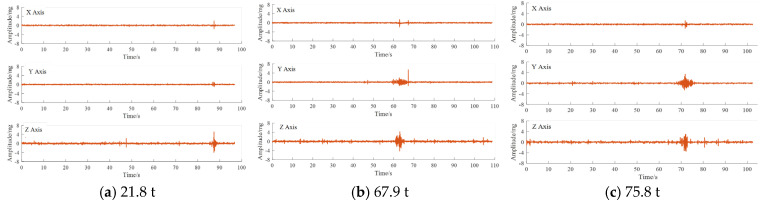
Power response data for different vehicle passages.

As can be seen from Figure 6, when the above vehicle passes through the monitoring point, the acceleration sensor produces a significant response in all three directions. Taking the data of a vehicle with a load capacity of 21.8 t as an example, the peak value of the vertical upward vibration response signal is 5.12 mg, the peak value of the horizontal vibration response signal in the X-direction is 1.97 mg, and the peak value of the vibration response signal in the Y-direction is 0.69 mg. It can be seen that the vibration signal in the vertical direction of is significantly larger than the remaining two directions. By analysing the pavement vibration data under different vehicle loads (21.8 t, 67.9 t, and 75.8 t), it can be found that the amplitude and duration of vibration show a significant upward trend with the increase in vehicle weight. The horizontal (*X*-axis) and longitudinal (*Y*-axis) vibrations exhibit relatively small amplitudes, in the range of ±1 mg and ±3 mg, respectively. In addition, the *Z*-axis signals contained more high-frequency components, especially during the vehicle passage period (60–80 s), indicating a strong correlation between vehicle weight and the dynamic response of the pavement. These findings provide valuable insights into the effects of heavy vehicle loads on the behaviour of pavement structures, which can be used for pavement infrastructure performance assessment.

**Figure 7:**
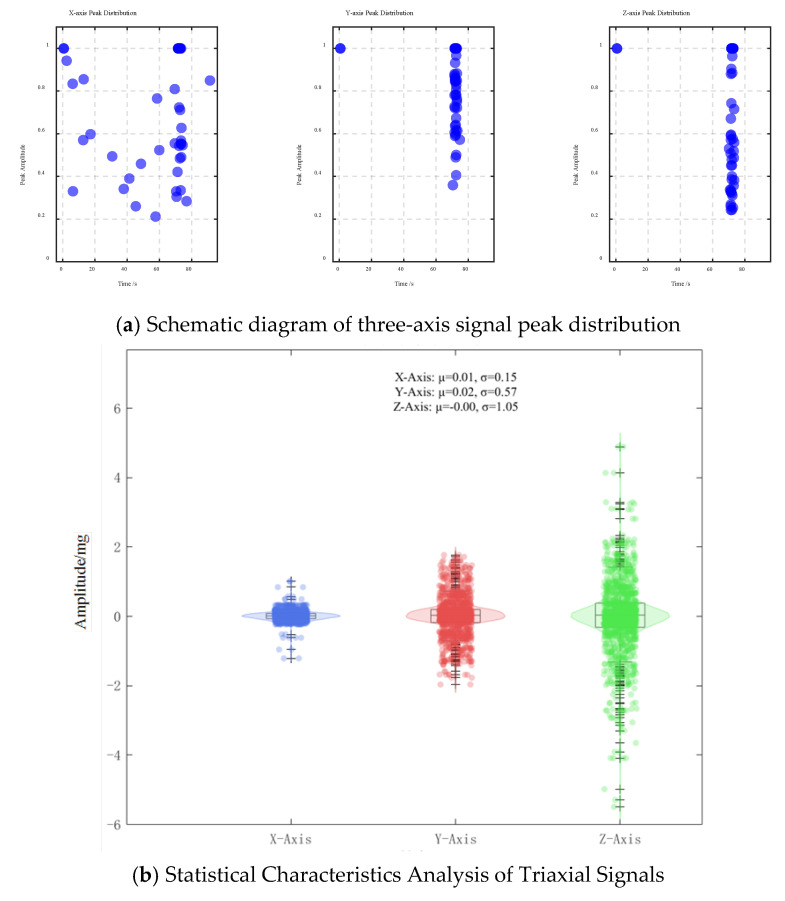
Three-axis peak distribution.

**Figure 8:**
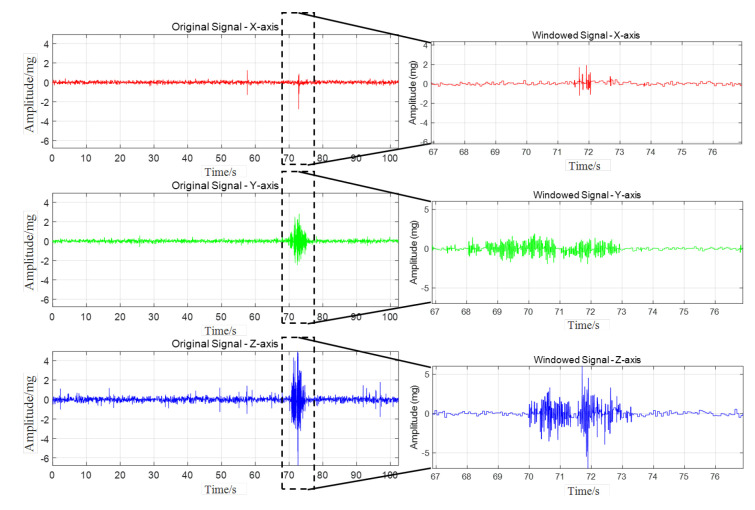
Extraction of three-axis power response data.

When the vehicle passes through the monitoring point, the acceleration sensor data peaks. In order to study the relationship between the peak values of vehicle and sensor data, the peak data of 20 sensors in the experiment were extracted. The peak distribution map of the three-axis acceleration is summarized in Figure 7. After the accurate time period of vehicle passage was located, data with a duration of 10 s was extracted for analysis. The corresponding acceleration response map of vehicle passage is shown in Figure 8.

Figure 7a shows the peak amplitude distribution of the acceleration signal along the X-, Y-, and Z-axes over time. The results show that the vibration peaks are sparsely distributed before 60 s but become more concentrated between 70 and 80 s, consistent with the vehicle passage time in the captured video. The peak amplitude of the Z-axis is significantly higher compared to those of the X- and Y-axes, indicating that vertical vibration dominates the road surface response. When the axle passes through the monitoring point, the road surface is excited by the tyre, producing transient deformation and displacement, so the acceleration sensor buried in the road surface will produce a peak response. However, from the sensor response shown in Figure 8, it can be found that the three-axis data cannot intuitively show the number of peaks matching the selected three-axis experimental vehicle. After the sub-experiment, it was found that the number of peaks does not match the axle type of the vehicle. Matching is common. On the whole, the main reasons for the mismatch between the number of peaks and axles are the complexity of sensor responses, signal attenuation and propagation effects, and the limitations of signal processing methods.

From a pavement mechanics perspective, the dominance of the *Z*-axis (vertical) vibration response is a direct consequence of the primary loading mechanism. The principal force exerted by a heavy vehicle is the high-magnitude dynamic load acting vertically downwards on the pavement. For a rigid structure such as a cement concrete slab, this concentrated vertical force induces localized flexural deformation and transient deflection, creating a strong stress wave. As the sensor’s *Z*-axis is aligned with this primary load vector, it captures this high-energy impact most directly and sensitively. In contrast, horizontal excitation forces are secondary effects. These include longitudinal forces from rolling resistance or braking (*X*-axis) and lateral forces from vehicle sway or tyre cornering (*Y*-axis). The magnitude of these horizontal forces is significantly smaller than the vehicle’s gravitational load and dynamic vertical impact. Consequently, the pavement vibration response stimulated by these secondary forces is naturally much weaker.

In summary, the predominance of the *Z*-axis response is a direct manifestation of the vertical vehicle load being the primary excitation source. This physical understanding provides a critical rationale for our subsequent analysis: the *Z*-axis signal contains the richest and highest signal-to-noise ratio (SNR) information related to axle passage, making it the most ideal signal source for accurate axle-type identification.

### 4.2. EMD-Based Triaxial Vibration Signal Characterization

To address the mismatch between the detected peak counts and the actual vehicle axle configuration, the next phase of analysis will focus on refining the signal processing methodology. Empirical mode decomposition (EMD) will be employed to decompose the raw acceleration signals into intrinsic mode functions (IMFs), enabling the extraction of effective vibration components associated with vehicle excitation. Subsequently, short-time energy analysis will be applied to the decomposed signals, allowing for the identification of distinct energy peaks corresponding to vehicle axles. By analysing the energy distribution patterns, a more accurate representation of axle-induced pavement responses can be obtained, improving the reliability of axle detection. This approach aims to enhance the interpretability of sensor signals and provide a robust method for vehicle-induced vibration characterization in pavement monitoring applications.

**Figure 9:**
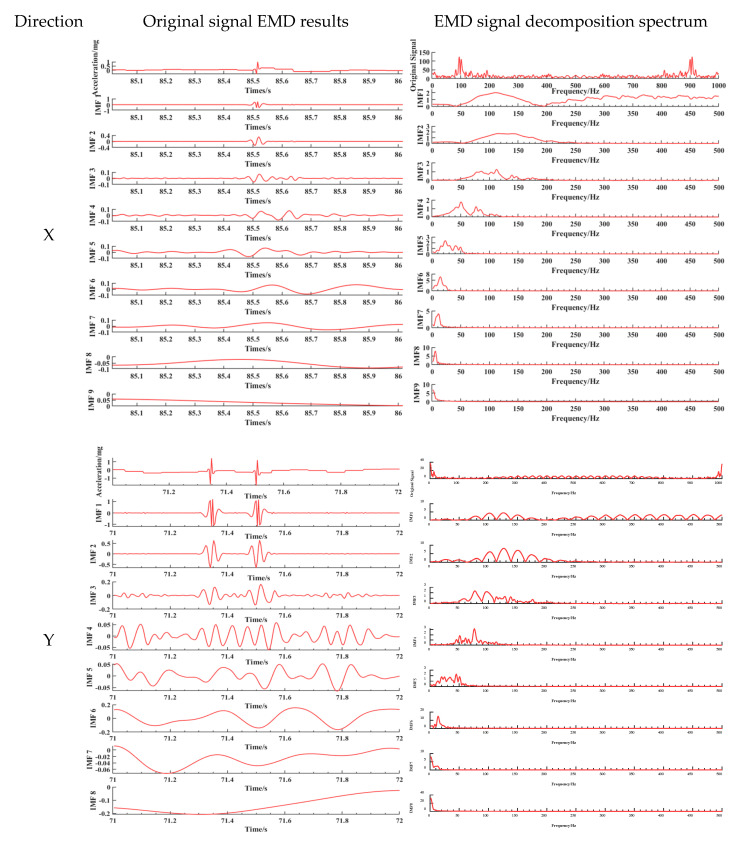

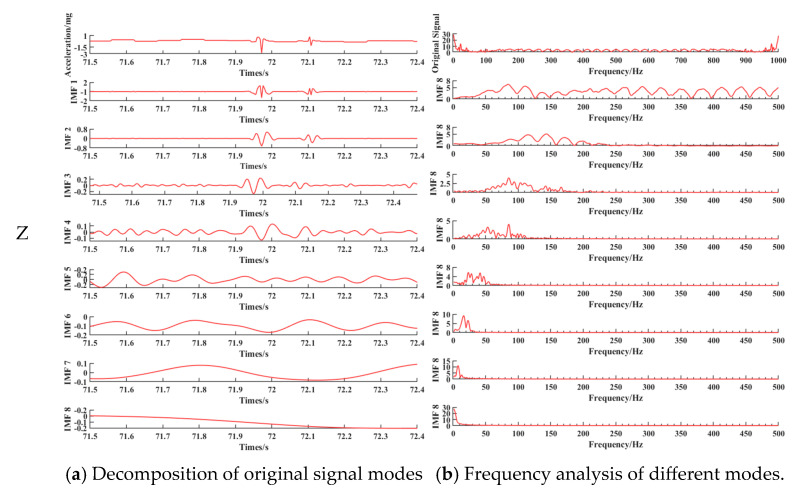
Vibrational signal decomposition and frequency analysis using the EMD.

Taking the road acceleration data collected when a three-axle truck with a load of 75.8 t and a speed of 20 km/h passes through in the above paper as an example, the data in three directions are decomposed, respectively, and the spectral characteristics of components in different decomposition modes are analysed. Figure 9 shows the results of the time–frequency analysis of vibration signal decomposition.

**Figure 10:**
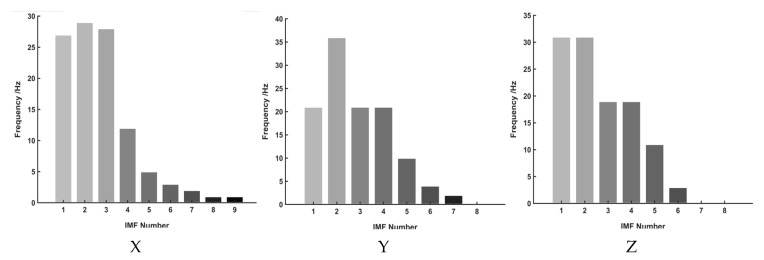
The EMD decomposition spectrum of each component of the three-axis vibration signal.

The empirical mode decomposition (EMD) of the triaxial vibration signals revealed a structured distribution of frequency components across the intrinsic mode functions (IMFs). Extensive research by predecessors has demonstrated that the interaction between vehicles and road surfaces generates a wide frequency band of excitation, with the high-frequency components (>100 Hz, even up to kHz levels) primarily caused by the structural dynamics of the vehicle body (such as tyre tread patterns and tyre body vibration) and the fine texture of the road surface [42,43,44,45,46,47]. In this paper, the spectral characteristics after signal decomposition are shown in Figure 10. For the *X*-axis, a clear trend of decreasing frequency is observed from IMF1 to IMF9. High-frequency components and noise were predominantly concentrated in IMF1–IMF3 (100–200 Hz and ~900 Hz), characterized by rapid oscillations and lower amplitudes. Mid-frequency components (50–150 Hz, peaking around 100 Hz) were evident in IMF2 and IMF3, while IMF4 and IMF5 contained primarily low-frequency content (<50 Hz). Lower-order IMFs (IMF6–IMF9) were dominated by near-zero frequencies, reflecting low-frequency trends and system influences.

The *Y*-axis exhibited significant high-frequency vibrations and strong amplitude fluctuations in IMF1–IMF3 (100–200 Hz). Frequency and amplitude progressively decreased with increasing IMF order, with IMF7–IMF9 representing smooth, low-frequency trends (<50 Hz). Mid-frequency components (50–150 Hz) were concentrated in IMF4 and IMF5.

On the *Z*-axis, IMF1–IMF3 contained high-frequency components with rapid fluctuations, with IMF1 displaying the highest amplitude and frequency (200–300 Hz). Frequencies and amplitudes decreased in IMF2 and IMF3. IMF4–IMF6 demonstrated progressively lower frequencies and more stable amplitudes (50–150 Hz), likely capturing primary structural vibrations. IMF7–IMF9 were characterized by low-frequency, slowly varying trends (<50 Hz), potentially reflecting long-term environmental influences.

### 4.3. IMF Filtering

To accurately extract vehicle-induced vibration signals and identify axle types, short-time energy (STE) analysis was performed on selected intrinsic mode functions (IMFs) obtained through empirical mode decomposition (EMD). The selection of IMFs is based on their energy distribution and frequency characteristics, ensuring the retention of key vehicle-induced dynamic responses while minimizing noise and irrelevant trends.

**Figure 11:**
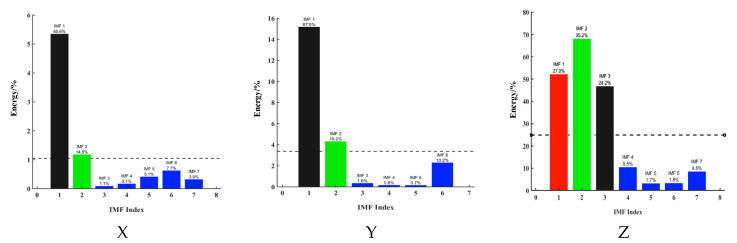
Modal energy proportion of three-axis road dynamic response signal under vehicle excitation.

The empirical mode decomposition (EMD) method was employed to analyse pavement dynamic response signals under vehicle excitation. Signal components induced by vehicle excitation were initially identified based on amplitude and frequency characteristics. To further validate the contribution of different modal components, short-time energy analysis was conducted to quantify the energy distribution across intrinsic mode functions (IMFs), as shown in Figure 11.

The results reveal significant differences in modal energy distribution across directions. In the X-direction, IMF1 exhibits the highest energy share (65.6%), followed by IMF2 (14.5%), while higher-order IMFs contribute negligibly, indicating dominance of lower-order modes. Similarly, in the Y-direction, IMF1 (67.5%) and IMF2 (19.2%) capture the majority of the energy, with higher-order IMFs contributing less than 2%, reinforcing the dominance of low-frequency components in pavement response. In contrast, the Z-direction shows a more balanced modal energy distribution, with IMF2 (35.2%), IMF1 (27.0%), and IMF3 (24.2%) collectively contributing most of the energy, while higher-order IMFs account for less than 13%.

A comprehensive analysis confirms that the vehicle-induced pavement response is primarily concentrated in the lower-order IMFs (IMF1 and IMF2), consistent with the low-frequency nature of vehicle excitation. Higher-order IMFs (IMF3 and above) contribute less than 20% of the total energy in the X- and Y-directions, indicating their limited role in overall dynamic response. Although the *Z*-axis exhibits slightly higher contributions from higher-order IMFs, their influence remains minor compared to the dominant low-frequency components. This suggests that vehicle excitation is predominantly low-frequency, with high-frequency components affecting only localized *Z*-axis responses. The modal distributions of the X- and Y-axes are highly similar, reflecting the in-plane consistency of vehicle-induced vibrations, while the *Z*-axis exhibits a more complex energy pattern, likely influenced by high-frequency excitation and road surface microtextures.

To achieve robust axle-type identification, short-time energy analysis was applied to the selected lower-order IMFs. These IMFs effectively preserve time-localized energy fluctuations inherent in vehicle excitation, aligning with dominant low-frequency dynamics such as suspension oscillations and tyre–road interactions. Higher-order IMFs, contributing less than 20% of the total energy, were excluded to mitigate interference from high-frequency noise and transient artifacts unrelated to axle signatures.

### 4.4. Vehicle Axle Type Recognition Based on Short-Time Energy Profile

During vehicle driving, the interaction between the tyre and the road surface produces a transient excitation signal, and the energy distribution pattern of this signal is different under different axle types. Through the short-time energy calculation of the IMF (intrinsic mode function) component, the effective signal related to vehicle axle type can be extracted, the high-frequency noise and environmental interference can be eliminated, and the axle type recognition can then be achieved.

After the short-term energy curve is calculated, the energy peak distribution of different IMFs is observed. Different axle types of vehicles produce different excitation modes, resulting in differences in the number of peaks and distribution patterns of short-term energy on the time axis. For example, a short-term energy peak of a two-axle vehicle typically exhibits two distinct pulses, while a three-axle or multi-axle vehicle exhibits a corresponding number of peaks. Based on the short-term energy curve, the number of peaks and their time intervals are counted and compared with known vehicle parameters. If the adjacent peak interval matches the vehicle wheelbase, the axle type corresponding to the signal can be confirmed. Finally, the applicability and stability of this method are verified by several groups of samples, and its ability to identify vehicle axle shapes under different working conditions is ensured.

In order to ensure the comparability of vibration signals measured by different sensors and improve the accuracy of short-term energy analysis, the processing and calculation methods in this paper are as follows:

(1) Signal normalization

Since different sensors may have sensitivity differences, the analysis using raw acceleration data may directly lead to bias in the results. Therefore, the vibration signal is firstly normalized to limit its amplitude range between [−1, 1] to eliminate individual sensor differences and improve data consistency.

(2) Sliding window segmentation

The sliding window technique is used to segment the signal for a short time to capture the local dynamic characteristics under vehicle excitation. The selection of STE parameters is mainly based on the physical size of the experimental road section and the dynamic characteristics of the vehicle. The monitoring points are arranged in a 4.5 m long cement concrete slab. Due to the particularity of this experimental section, the speed limit of transport vehicles was 30 km/h (about 8.33 m/s). The wheelbase range for heavy goods vehicles travelling on this section is usually between 1.3 m and 2.0 m. The duration of a single axle impact event is very short, while the time interval of adjacent axles passing through the sensor can be calculated to be about 0.16 s to 0.24 s. Therefore, 0.2 s is selected as the window length to ensure that the energy of axle impact can be completely captured, and at the same time, the energy peaks of adjacent axles can be effectively distinguished, so as to avoid the problem of peak merging caused by a window that is too long. The window range (0.1–0.5 s) given in the paper covers the transit time of all vehicles. Frame shift (R) adopts an overlap rate of 50% according to the common setting in signal processing (that is, frame shift R = 0.1 s) to ensure continuity between frames and avoid missing transient events at the edges of the window.

(3) Spectrum leakage suppression

A Hamming Window is applied on each short-time frame, and its mathematical expression is as follows:

(13) $$w\left(n\right)=0.54-0.46\cos\left(\frac{2\pi n}{N-1}\right)\;0\leq n\leq N-1$$

where N is the window length, and n is the current time point. The Hamming window can effectively reduce spectrum leakage and improve the accuracy of short-term energy calculation.

(4) Short-term energy calculation

The short-term energy is calculated for each frame signal to reflect the instantaneous vibration intensity. Based on the aforementioned energy distribution analysis, lower-order IMFs (such as IMF1 and IMF2) are selected as the main research objects to ensure that the key features of vehicle excitation are retained. Short-time energy calculation is performed on selected IMF components, and its mathematical expression is as follows:

(14) $$E\left(n\right)=\sum_{m=0}^{N-1}x^{2}\left(n-m\right)w\left(m\right)$$

where E(n) is the short-time energy at the nth time, x(n) is the signal sequence, w(m) is the sliding window function, and N is the window length.

**Figure 12:**
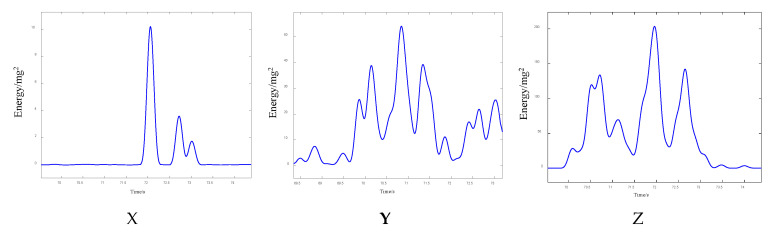
Three-axis short-term energy distribution characteristics of cement concrete pavement under vehicle-mounted excitation.

For the above three-axis acceleration sensors, the short-time energy of the data in the X-, Y-, and Z-directions is calculated as follows. The final three-axis short-time energy line graph is shown in Figure 12 below:

Figure 12 illustrates the temporal distribution of short-term energy (STE) derived from tri-axial sensor data (X-, Y-, and Z-axes) during a vehicle passage event between 69.5 s and 74.5 s. Distinct response patterns are observed across the axes:

*X*-axis (longitudinal/transverse): energy peaks are sharp and localized, especially around 71.8 s and 72.6 s, with low energy levels between peaks, indicating discrete axle impacts.

*Y*-axis (transverse/longitudinal): the energy profile is broader, with higher baseline levels, showing peaks around 70.2 s, 71.1 s, and 71.8 s, the latter coinciding with the *X*-axis peak. These peaks are less defined.

*Z*-axis (vertical): Significant fluctuations occur, with peaks around 70.4 s, 71.1 s, 71.8 s, and 72.5 s. These broader peaks may result from both vertical loading and structural vibrations.

Together, these STE profiles reveal axis-dependent energy bursts indicative of axle passage, reflecting the anisotropic nature of sensor responses and stress wave propagation in the structure.

Despite these insights, direct axle identification remains difficult due to noise and other sources of interference, which causes discrepancies between the number of detected peaks and actual axle types.

In order to overcome the problems described above, this section improves the accuracy and stability of identifying axles from STE signals by setting three indicators: minimum peak height, minimum peak protrusion, and minimum peak distance.

(1) Minimum peak height: The amplitude of energy signals generated by different vehicles (no load/heavy load) and different axles (steering shaft/drive shaft) is quite different, and the signal baseline may fluctuate, so the fixed threshold is not effective. Therefore, this paper adopts an adaptive strategy based on the statistical characteristics of signals. The STE threshold is determined based on the global maximum peak value. First, find the global maximum peak on the short-time energy (STE) curve obtained. Then, by analysing the noise level, the height threshold for peak detection is set at 30% of the maximum peak value. This method can automatically adjust the threshold based on the overall energy level of the signal, ensuring that significant energy peaks caused by shaft impacts can be effectively distinguished from background noise and minor fluctuations. This approach avoids the bias that might be introduced by using a fixed absolute value threshold.
(2) Minimum peak prominence: This parameter is crucial for distinguishing independent true axle peaks from shoulder peaks on the main peak or secondary energy peaks caused by vibration. It quantifies the significance of a peak with respect to its surrounding signal, requiring that an effective peak must have sufficient relative height within its local neighbourhood. In this paper, through empirical iterative optimization of experimental data, it was found that when the minimum peak outburst factor is set to 0.4, most interference peaks (such as fluctuations on wide peaks) caused by non-axle load can be effectively filtered out, while two independent peaks that are close to each other but do exist within a parallel axle group can be retained. This value, in the range of 0.2 to 0.6, ensures maximum detection specificity without loss of true axle peaks, particularly the second axle in a parallel axle configuration.
(3) Minimum peak distance: Set a physically reasonable minimum peak time interval (minimum axle time interval) based on the minimum wheelbase and expected traffic speed of the vehicle. This constraint (minimum peak distance sample) prevents signal oscillations or broadening peaks caused by a single axle impact from being incorrectly identified as multiple adjacent axles, ensuring physical plausibility of the detection results. Most of the experimental vehicles studied in this paper were low-speed heavy trucks, and their wheelbase was usually between 1.3 m and 2.0 m. Combined with the fact that the vehicle speeds in this study were all lower than 30 km/h, the theoretical shortest transit time between axles could be calculated to be about 0.144 s. In order to ensure robustness and leave a certain margin, the minimum axle time interval was set as 0.15 s in this study, which ensured that adjacent axles could be effectively distinguished even in the limiting cases of maximum speed and minimum wheelbase.

**Figure 13:**
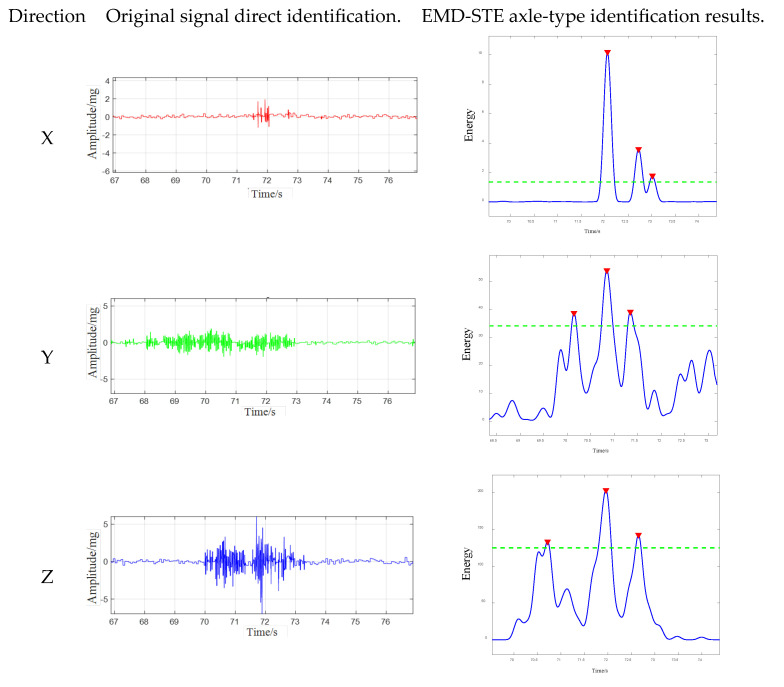
Comparison of Vehicle Axle Type Recognition Effect.

In order to directly demonstrate the effectiveness of the method proposed in this paper, Figure 13 lists the comparison between the original signal and the axle pattern recognition effect processed by the EMD-STE method. This figure shows the clear results after processing the same original signal segment. The original signal segment contains many peaks, making it difficult to directly identify the vehicle axle pattern, and the number of peaks makes it difficult to directly match the vehicle axle pattern. After processing using the method proposed in this paper, the number of peaks obviously matches the number of axles of the vehicle. In the result diagram of vehicle axle type identification using the EMD-STE method, the blue trajectory depicts the time curve of this STE signal. The dashed green line represents the minimum peak height threshold employed by the peak detection algorithm, and only STE peaks exceeding this amplitude are considered potential axle events. Three distinct peaks in the STE signal exceed the defined threshold. These detected events are indicated by red inverted triangles, and the actual working condition is the passage of three axles of the vehicle. The STE analysis, combined with a threshold-based peak detection strategy, successfully identified three different energy events in the signal, corresponding to the expected number of axles of the target vehicle. The axle-related STE peaks appear to be significantly higher than the baseline energy level, indicating a good signal-to-noise ratio in the STE domain for this particular event. This helps to achieve reliable threshold processing. This direct comparison before and after processing illustrates that the method proposed in this paper effectively solves the core challenges, such as noise interference and peak adhesion in the original signal.

Ideally, when the centreline of a single axle passes directly above the sensor, the response peaks in each axial direction should occur simultaneously. However, the actual observed time difference is ubiquitous, and the main reasons can be attributed to the following points:

(1) Complexity of vehicle–sensor interaction: When the wheel comes into contact with the road surface and passes through the sensor area, the force/vibration generated is not an ideal instantaneous pulse, but a dynamic process. The rolling, deformation, and slip of the tyre, as well as the compression and rebound of the suspension system, will produce dynamic responses with different phase and timing characteristics in the vehicle travel direction (X), lateral direction (Y), and vertical direction (Z). For example, the vertical force (*Z*-axis) may peak closest to the wheel centre point through the sensor location, while the longitudinal force (*X*-axis) may peak slightly earlier or later due to rolling resistance or drive/braking torque. The peak of the lateral force (*Y*-axis) may be related to the slight lateral sloshing of the vehicle or the tyre cornering characteristics.
(2) Signal propagation and processing: There may be differences in the propagation path and speed of vibration signals inside the road structure or sensor. In addition, short-time energy calculation involves window function and integration/averaging process, which itself may introduce a small time delay, and the difference in signal waveform in different axial directions will cause the calculated energy-peak time point to shift.
(3) Dynamic behaviour of the vehicle: Dynamic factors, such as vehicle speed, uneven load distribution, tyre pressure, and attitude change (pitch, roll) when crossing hurdles will complicate the time series relationship of the force generated by axles on sensors in three-dimensional space, resulting in incomplete synchronisation of the peak energy in each axial direction.

**Figure 14:**
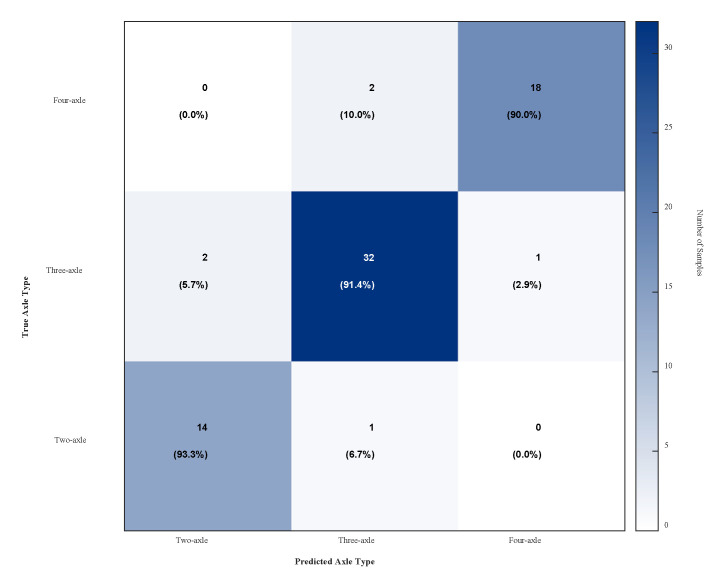
Confusion matrix of axle identification results based on the EMD-STE method.

For a quantitative evaluation of the EMD-STE-based axle identification algorithm, we applied it to the complete set of 70 experimental datasets and compared the results with the ground truth obtained from synchronised video recordings. Figure 14 presents the detailed confusion matrix. The rows of the matrix represent the true axle type of the vehicles, while the columns represent the predicted type by the algorithm. The values within each cell display both the sample count and the row-normalized percentage.

As can be clearly seen from Figure 14, the algorithm demonstrates excellent identification performance. Of the 15 two-axle vehicles, 14 were correctly identified (93.3% recall); of the 35 three-axle vehicles, 32 were correctly identified (91.4% recall); and of the 20 four-axle vehicles, 18 were correctly identified (90.0% recall). This method performs well on this data set but is limited by experimental conditions. The number of experiments carried out is still relatively small and will continue to be carried out in the future.

This study employs short-term energy analysis of triaxial (X, Y, Z) vehicle traffic signals to identify axle types. Successful detection of three significant energy peaks confirmed the method’s effectiveness in identifying a three-axle vehicle. However, observed time discrepancies between energy peaks from the same axle across axes, a common phenomenon in multi-axis systems, highlight the complexity of three-dimensional vehicle–sensor interaction. While not affecting axle count accuracy in this instance, this timing variability necessitates further investigation for applications requiring precise wheelbase measurement or multi-axis data fusion. Future work will focus on developing algorithms that leverage this temporal information for enhanced vehicle characterization.

### 4.5. Comparison with Existing Vehicle Detection Technologies

The core advantage of the vehicle detection method (MEMS + EMD-STE) proposed in this paper lies in cost-effectiveness. MEMS acceleration sensors benefit from the huge scale effect of the consumer electronics industry, and their unit price has been significantly reduced, providing economic feasibility for large-scale and high-density deployment in road networks. Moreover, the sensor is small in size, and the embedded installation mode causes relatively little disturbance to the pavement structure. As a solid-state device without mechanical moving parts, it has the characteristics of high reliability and low maintenance requirements. Modern MEMS sensors are designed with good electromagnetic compatibility, and through appropriate shielding design, they can effectively resist common environmental electromagnetic interference. Traditional methods can only provide single-dimensional physical quantities (such as peak strain and magnetic field change), but the three-axis acceleration sensor used in this paper can obtain rich three-axis dynamic response data. Through advanced signal processing technologies such as EMD-STE, we can extract energy characteristics closely related to axle impact from complex vibration signals, which can theoretically achieve higher axle-type recognition accuracy compared with simple peak detection of the original signal when dealing with complex traffic flows such as low speed, multi-axle, and similar wheelbase. More importantly, these rich dynamic data provide technical support for extracting vehicle speed and road status in the future.

In order to better assess the application value of the “MEMS + EMD-STE” method proposed in this study, this paper summarizes the advantages and disadvantages of the existing vehicle detection technologies based on a literature review and compares them with the method proposed in this paper across key performance dimensions. The results are shown in Table 3.

In summary, the “MEMS + EMD-STE” method proposed in this study demonstrates a unique, balanced advantage among numerous technologies: it leverages low-cost, high-robustness hardware (MEMS) and combines advanced signal processing algorithms. The aim is to achieve richer and more precise functionality than traditional low-cost detection technologies (such as loop detectors) at a significantly lower cost than that of high-end WIM systems (such as piezoelectric, fibre optic) and so on.

## 5. Conclusions

This research focused on characterizing the triaxial dynamic response of cement concrete pavement under low-speed, heavy-duty vehicle loads, utilizing field monitoring data and advanced signal processing techniques. A pavement vibration monitoring system with embedded accelerometers was successfully implemented to acquire response signals under operational traffic. Based on the analyses performed, the main conclusions are as follows:

(1) Low-speed, heavy-duty vehicles generate significant dynamic responses along all three measurement axes. The vertical (*Z*-axis) vibration is consistently dominant, showing the largest amplitudes and higher principal frequency content compared to the horizontal (X- and Y-) directions. This underscores the primary role of vertical impact dynamics in pavement response under these loading scenarios.
(2) Empirical mode decomposition proved effective in dissecting the complex, non-stationary vibration signals into a series of intrinsic mode functions (IMFs). This decomposition revealed distinct frequency bands within the signal: high-frequency oscillations (IMF1-3) linked to transient vehicle impacts, mid-frequency components (IMF4-6) potentially reflecting structural and vehicle dynamic interactions, and low-frequency variations (IMF7-9) associated with underlying trends or noise. The *Z*-axis signal generally exhibited richer high-frequency content.
(3) Short-time energy (STE) analysis, particularly when applied to the lower-order IMFs identified through EMD, effectively captured the transient nature of vehicle axle passages. These events manifested as distinct, sharp peaks in the STE profile, confirming the impulsive characteristic of axle loads. The analysis indicated that the primary energy from vehicle excitation is concentrated within these transient events and largely contained within the initial IMFs.
(4) While identifying axle configurations directly from raw or simply filtered signal peaks proved challenging due to signal complexity and noise, the sequential application of EMD (to isolate relevant signal components) and STE analysis (to identify energy bursts) provides a more robust foundation. By applying optimized peak detection criteria (considering minimum height, prominence, and distance) to the STE profiles derived from selected IMFs, a more reliable method for axle event detection can be established, paving the way for accurate axle counting and classification.

EMD and STE have shown great potential in analysing road vibration data caused by low-speed and heavy-duty vehicles, but there are still some limitations in the current research. In future research, more robust signal decomposition technologies, such as CEEMDAN, will be introduced to overcome the modal aliasing limitations of current EMD methods, and the application of deep learning models such as CNN and RNN will be explored to build an end-to-end intelligent recognition framework to improve the accuracy and generalization ability of the algorithm. Secondly, the research will shift from single-point analysis to multi-sensor spatiotemporal information fusion and make full use of sensor array data through cross-correlation analysis and other methods, not only to accurately estimate vehicle speed, but also to track the propagation characteristics of vibration waves. This, in turn, deepens the understanding of vehicle–road coupling mechanism and explores the potential of identifying localized defects on road surfaces. On this basis, the application goal will expand from axle-type recognition to more challenging functions, including establishing a quantitative model between vibration energy and vehicle axle load for dynamic weighing (WIM) and using the time-difference information of three-axis signals to evaluate the vehicle trajectory. Finally, in order to ensure the engineering practicability of the method, future research must systematically model and analyse the influence of real environmental and operational factors, such as temperature, humidity, vehicle speed change, and road aging on the monitoring signal, so as to establish an all-weather, highly robust monitoring system that is capable of adaptive compensation.

## Figures and Tables

**Table 1:** Road structural parameter.

Structure Name	Thickness/cm
Cement concrete surface layer	30
AC-10F asphalt concrete stress-absorbing layer	3
PC-2-modified emulsified asphalt permeable layer	—
5% cement-stabilized gravel subbase	18
5% cement-stabilized crushed stone base course	18
Subgrade improvement layer	18

**Table 2:** Vehicle data from the monitoring point.

No	Axis Weight/t	Vehicle Shaft Type	Vehicle Photos
1	2.10	Two-axis	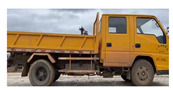
2	18.3	Four-axle	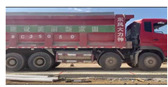
3	21.8	Three-axis	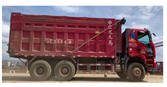
4	57.1	Three-axis	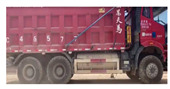
5	66.9	Three-axis	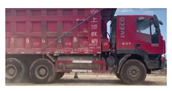
6	67.9	Three-axis	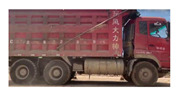
7	72.1	Three-axis	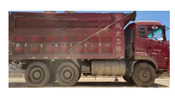
8	73.8	Three-axis	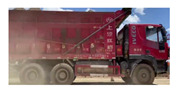
9	75.7	Three-axis	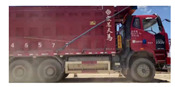
10	75.8	Three-axis	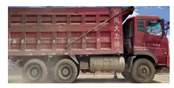

**Table 3:** Comparison of existing vehicle detection methods and technologies.

Technology	Cost and Scalability	Ease of Installation and Maintenance	Anti-Electromagnetic Interference Capability	Theoretical Accuracy and Functionality Richness
Piezoelectric	High: sensors and backend devices are expensive.	Low: high requirements for the installation process, requiring regular calibration or replacement.	Medium, temperature sensitive	High, high weighing accuracy, but other functions are limited
Fiber Optic	Extremely high: both the sensors and the demodulation equipment are very expensive.	Moderate in quality, with high installation requirements, but the sensor itself is stable.	Extremely high, fully immune to electromagnetic interference	High, high precision, but cost-limiting applications
Inductive Loop	Low: mature technology, low cost.	Low: installation damages the pavement, easily damaged under stress.	Low, highly susceptible to interference from electromagnetic sources	Low, only capable of detecting the presence of vehicles, with limited functionality, and at risk of missing detections
Methods of this study (MEMS + EMD-STE)	Low cost: MEMS sensors are suitable for large-scale deployment.	High: embedded installation, low disturbance, high reliability of solid-state equipment.	High, designed to effectively resist interference	High, capable of obtaining tri-axis dynamic data, with rich features and great potential for axle-type identification

## Data Availability

The original contributions presented in this study are included in the article. Further inquiries can be directed to the corresponding author(s).

## Abbreviations

The following abbreviations are used in this manuscript:

STE	Short-term energy analysis
EMD	Experience Mode Decomposition
